# Improved Detection of Polysulfated Oligosaccharides by Mass Spectrometry Applicable to Miniaturized Samples

**DOI:** 10.3390/molecules29235642

**Published:** 2024-11-28

**Authors:** Frédéric Jeanroy, Julie Gil, Clothilde Comby-Zerbino, Claire Demesmay, Vincent Dugas

**Affiliations:** 1 Universite Claude Bernard Lyon 1, ISA, UMR 5280, CNRS, 5 Rue de la Doua, 69100 Villeurbanne, Francejulie.gil@univ-lyon1.fr (J.G.); demesmay@univ-lyon1.fr (C.D.); 2 Universite Claude Bernard Lyon 1, ILM, UMR 5306, CNRS, 69100 Villeurbanne, France; clothilde.zerbino@univ-lyon1.fr

**Keywords:** nanochromatography, glycosaminoglycan, MALDI-TOFMS

## Abstract

The study of biomolecules and their interactions in their natural environment requires increasingly sophisticated technological and methodological developments. The complexity of these developments is due, among other things, to the nature of these molecules and the small quantities available depending on their origin. In this context, this study focuses on the conditions for improving the detection of glycosaminoglycans on a miniaturized scale by mass spectrometry. These multicharged anionic linear polysaccharides are in fact difficult to study by mass spectrometry and can present, for a given molecule, a large number of signals linked to different charge states, to the loss of one or more sulfate groups and to the presence of different adducts, which reduces sensitivity and complicates the interpretation of the spectra. In order to reduce this complexity, we have investigated different sample preparation methods applicable to small sample volumes. The development of home-made capillary ion-exchange columns, for example, makes it possible to control the adducts formed in nano-ESI coupling. However, their use on a miniaturized scale for detection by MALDI-TOF-MS does not allow for performances as high as those obtained with treatment with a commercial DOWEX^TM^ resin. However, experimental results allowed us to demonstrate that the presence of DOWEX^TM^ resin colloid residues in the aqueous phase greatly improves the quality of the spectra obtained by MALDI-TOFMS on a Fondaparinux model glycosaminoglycan.

## 1. Introduction

**Scheme 1:**
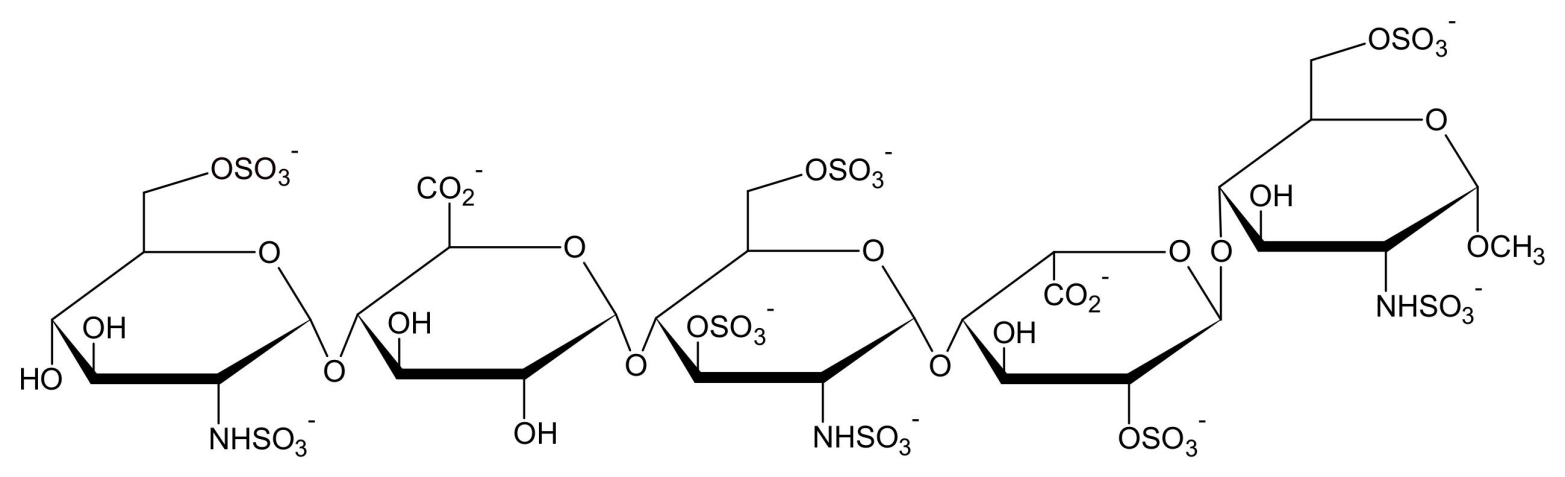
Chemical structure of the synthetic Fondaparinux pentasaccharide [GlcNAc6S−GlcA −GlcNS3S6S−IdoA2S−GlcNS6S].

Glycosaminoglycans (or GAGs) are multi-negatively charged linear polysaccharides of the extracellular matrix involved in a large number of biological processes [1,2]. Their study and more particularly the identification of their site of interaction with cellular partners is very challenging, requiring us to push the limits of analytical chemistry [3,4]. In this context, mass spectrometry is an essential tool for detecting, identifying, and analyzing these molecules. Its coupling with separation tools (such as chromatography or capillary electrophoresis) makes it possible to reduce the complexity of mixtures and contributes to the power of this tool. The mass spectrum for a compound is most often associated with a set of signals coming from the different forms (adducts, loss of sulfates, and in-source fragmentation) and the multiple charge states detected. Negative mode ionization (ESI, nano-ESI, and MALDI), because it presents very good ionization efficiency for these molecules [5,6], is the most suitable and most used mode for this type of compounds. Controlling the voltage applied to the ionization source can limit, or even eliminate, in-source fragmentation [5,6]. Even if GAGs can be detected in an intact form, the formation of various adducts leads to the splitting of the total signal intensity into several signals and to a decrease in detectability. These different forms, associated with the formation of ions of multiple charge states (in ESI for example), lead to a loss of sensitivity, the signal of a compound being distributed over several ions, and to complexity in the interpretation of the associated spectra. This is particularly evident for mixtures of depolymerized heparin such as Enoxaparin or low-molecular-weight heparin (LMW heparin). This type of complexity is also easily observed with the spectrum of a pure pentasaccharide Fondaparinux (Scheme 1), whether in nano-ESI-TOF or in MALDI-TOF-MS (Supplementary Material Figure S1).

The advantage of MALDI ionization is that it produces predominantly single-charged species, resulting in less complex spectra that are easier to interpret [7] and, therefore, more effective for high molecular masses. However, GAGs are likely to fragment more easily in the source due to the higher ionization energy than in ESI. Furthermore, ionization by MALDI cannot be coupled online with a chromatographic technique. Finally, even if they can also have the advantage of providing structural information during MS/MS analyses, many adducts are also likely to form with the matrix or alkaline metal ions (Na^+^, K^+^…), thus reducing the sensitivity. It is, therefore, often necessary to include a desalting step in the sample preparation. This step enables the saturation of the compounds with a chosen cation and the formation of preferential adducts. For example, to reduce the desulfation of GAGs, it is preferable to work with persodiated species [8].

Simplifying the mass spectrum and favoring certain adducts can, therefore, prove to be an essential step in order to unequivocally identify the compounds thanks to their exact mass. In order to favor a specific counterion, several strategies have been proposed. Cation-exchange resins, for example, introduced into a column or used in their solid form directly in the sample, have been used for several decades. Likewise, ion-exchange chromatography can be used as an intermediate step before analysis by mass spectrometry. The preparation of columns filled with a monolithic stationary phase functionalized with ionic groups on a miniaturized scale have even been described since the 2000s [9,10]. These two strategies are the most popular, but alternatives have also been proposed such as the use of sulfonated fluoropolymer (Nafion) or the use of crown ether capable of forming complexes with specific cations [11,12].

Due to the generally low amounts of available GAGs which furthermore cannot be overexpressed or amplified like other biopolymers [6], the development of an analytical method on a miniaturized scale is of great interest. In addition, to make it possible to target GAG analysis in a particular cell compartment, miniaturization would be a veritable asset in order to ultimately elucidate new specific binding domain sequences in the development of novel GAG-based therapeutics [13,14]. The objective of this work is to define a GAGs sample processing methodology, applicable on a miniaturized scale, to control the ionization states in order to reduce the complexity of the spectra and increase the sensitivity. Several strategies to control the ionization states of oligosaccharides have therefore been explored, with detection first by nano-ESI-MS and then by MALDI-TOFMS. Fondaparinux was chosen as a model GAG with the aim of favoring adducts formed with only sodium or only potassium which are also known to protect GAGs from desulfation phenomena.

## 2. Results and Discussion

One of the aims of sample preparation is to favor sodiated species in order to avoid desulfation and the observation of different adducts, which dilutes the signal and increases the complexity of the interpretation of the spectra. The use of a commercial ion-exchange resin (DOWEX) was proposed by Lesur et al. in 2019 for the analysis of oligosaccharides, including Fondaparinux, by MALDI-TOF-MS [15].

To adapt this strategy for the sample treatment of a miniaturized sample prior to nano-ESI-MS analysis, the preparation of ion-exchange nano-columns was first considered. The column must be able to exchange the counterions of the GAGs with sodium and should be able to be coupled on-line with the MS to be applicable to small samples.

In order to verify the usefulness of treatment with an ion-exchange resin, we first reproduced the sample treatment reported in [15]. The ion-exchange resin used in this example was a Dowex^TM^ resin prepared by the polymerization of styrene and divinylbenzene and functionalization with a sulfonate group in the presence of sulfuric acid. The nano-ESI analysis of Fondaparinux treated with the ion-exchange resin used in this example leads to (i) an almost complete absence of potassium adducts in the mass spectrum for the sodium-loaded resin and (ii) an almost complete absence of sodium adducts for the potassium-loaded resin, in contrast to the mass spectra obtained without any treatment (see Supplementary Material Figure S2).

Although very efficient, this resin cannot be used on-line as part of a nano-ESI-TOF or MALDI-TOF analysis coupled with nanochromatography, where sample volumes of a few microliters at most are available. In fact, resin bead diameters of 100–200 µm are too large to be packed on a miniaturized scale (e.g., capillary column). Therefore, other chromatographic ion-exchange strategies were considered.

### 2.1. Online Strategy with a Strong Cation-Exchange Monolith and Nano-ESI-MS

Two strong cation-exchange monolithic capillary columns were prepared from home-made poly(glycidyl methacrylate-co-ethylene dimethacrylate) monolithic capillary columns (75 µm i.d.) following two previously reported synthesis and grafting methods [16,17]. The first monolith was grafted with sodium bisulfite (Method A) and the second one with sodium mercaptoethane sulfonate (SMES) (Method B) (Supplementary Material Figure S3). The ion-exchange capacities of the monoliths were evaluated as described in the Section 3. The two ion-exchange capacities are comparable with exchange capacities of 0.24 ± 0.03 neq cm^−1^ (6.2.10^3^ ± 0.7.10^3^ neq mL^−1^) and 0.19 ± 0.03 neq cm^−1^ (5.0.10^3^ ± 0.8.10^3^ neq mL^−1^) for the column prepared with sodium bisulfite (Method A) and for the column prepared with sodium mercaptoethane sulfonate (Method B) respectively

**Figure 1:**
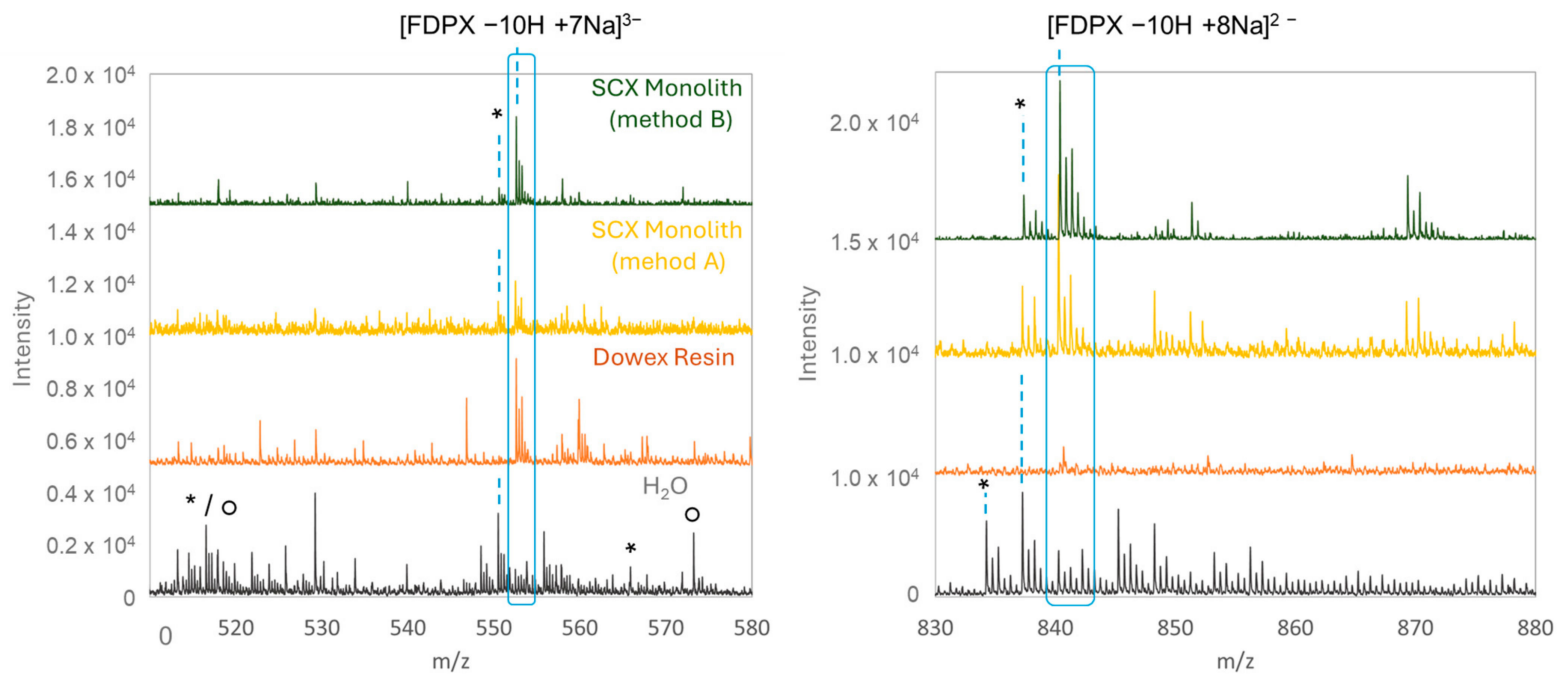
Mass spectra of a Fondaparinux solution (50 µM) obtained by nano-ESI-TOF during the infusion of solution at 0.4 µL/min with additional liquid methanol at 0.4 µL/min after elution from a monolithic exchange column of cations prepared from sodium mercaptoethane sulfonate (L = 8.5 cm, di = 75 µm), sodium bisulfite, or pretreated on Dowex^TM^ resin. The spectra were obtained in negative polarity, at a source voltage of 1.4 kV and a fragmentor voltage of 300 V. The asterisks represent the Na/K exchanges, and the circles represent the sulfate losses.

A Fondaparinux solution prepared in water was percolated through each of these two monolithic cation-exchange columns previously saturated with sodium ions and on-lined coupled with the mass spectrometer (Nano-ESI-MS). The mass spectra of Fondaparinux obtained after this on-line sample pretreatment (with both ion-exchange nano-columns) are shown in Figure 1 and compared with the spectrum obtained without pretreatment (FDPX in water) and with off-line pretreatment with the Dowex^TM^ resin. Regardless of sample pretreatment (Dowex^TM^ resin or monolithic ion-exchange columns), fewer adducts are detected than without pretreatment. Multiply charged ions are mainly detected (with different proportions for the di- and tri-charged ions depending on the sample pretreatment), and their major forms are mostly saturated with sodium ([FDPX − 10Na + 7Na]^3−^ and [FDPX − 10Na + 8Na]^2−^) or potassium ([FDPX − 10Na + 7K]^3−^ and [FDPX − 10Na + 8K]^2−^). These results demonstrate the efficiency of the pretreatment step compared to Fondaparinux without pretreatment (spectra in water Figure 1), with the absence of fully sodiated species and the presence of species with high sulfate losses.

During ionization, the K or Na adducts may be favored depending on the ion (potassium or sodium) chosen to saturate the ion-exchange column prior the sample pretreatment. In addition, very few Na/H or Na/K exchanges are observed. However, surprisingly, it seems here that the −3 charge state is favored after the pretreatment on the monolithic column compared to the spectra obtained after passing through the resin. Indeed, although still detected, the −2 state of charge is nevertheless much less intense.

This study of the pretreatment of Fondaparinux as a model GAG using miniaturized ion-exchange columns for nano-ESI ionization shows that it is possible to better control the species produced at the source (by favoring persodiated ions) and to limit the loss of the sulfate group. These promising results, therefore, led us to investigate the potential utility of this pretreatment for MALDI ionization.

### 2.2. Off-Line vs. On-Line Strategy with a Strong Cation-Exchange Monolith and MALDI-TOF-MS

These strategies (strong cation exchange with Dowex^TM^ resin or monolithic ion-exchange columns) have therefore been applied for the analysis by MALDI-TOF, which can be a technique of choice for the detection of oligosaccharide sequences on a miniaturized scale, for example after release from an affinity column [13]. The preferred counterion is sodium, as the MALDI-TOF spectrum shows that the sodium form of Fondaparinux is already in the majority without any pretreatment.

- Starting point: off-line strategy using FDPX pretreatment on Dowex^TM^ resin

**Figure 2:**
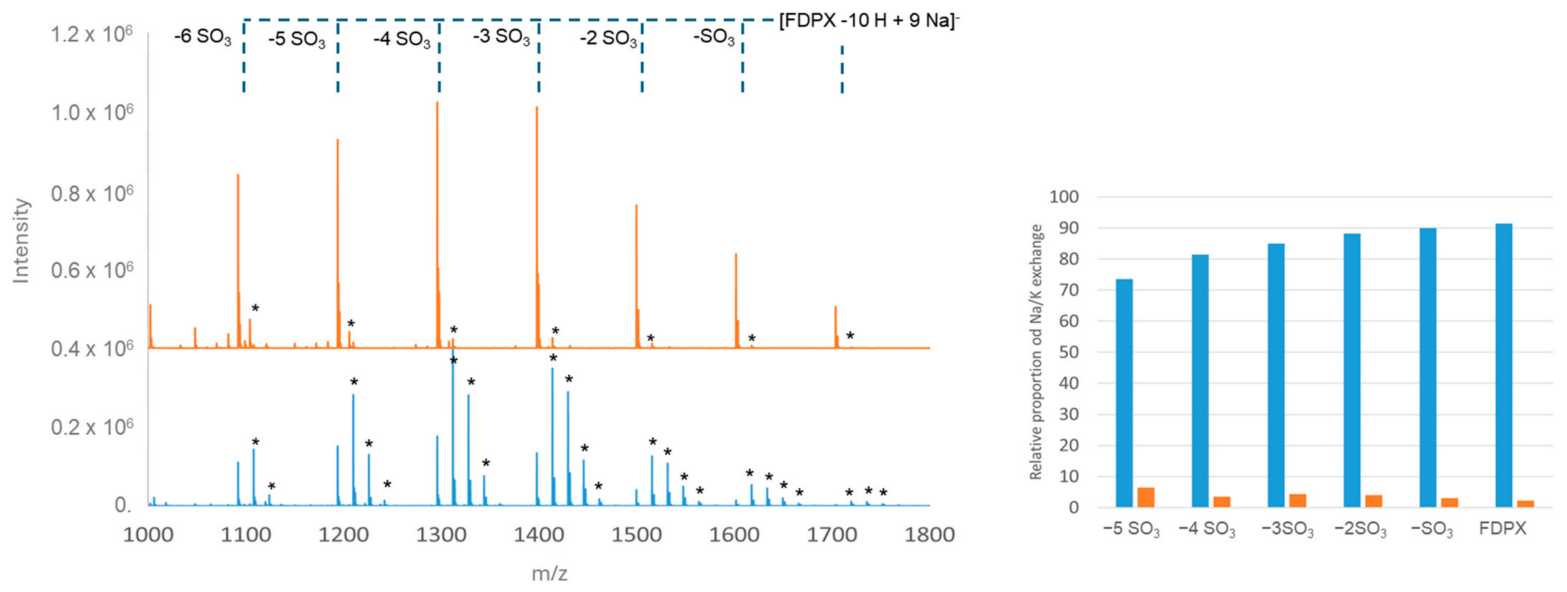
Mass spectra obtained by MALDI-TOF of Fondaparinux (50 µM) and proportion of forms presenting at least one Na/K exchange prepared in water (blue line) and after saturation with sodium ions by pretreatment on a DOWEX cation-exchange resin converted to Na (orange line); centered between *1000* and *1800 Th*. Samples are prepared with a HABA/TMG ½ 80 mg/mL matrix in methanol at a ratio of 0.5/1 µL of matrix/sample on a MALDI Anchorship plate. The spectra were obtained with a laser power of 73% for 100,000 spectra. Asterisks represent Na/K exchanges.

The use of a cation-exchange resin seems to prevent Na/K exchange even on the MALDI-TOF plate, according to previous results presented by Lesur et al. [15] The off-line use of this resin was therefore used as a reference point to study the influence of ion-exchange pretreatment of oligosaccharide sequences on the signal obtained by MALDI ionization. For this, the resin was first saturated with sodium ions using a 1 M NaOH solution (see Materials and Method), before being directly added to the Fondaparinux solution. Before the Fondaparinux solution was applied to the matrix, the resin was separated by centrifugation. Figure 2 shows the results obtained before (blue trace) and after treatment (orange trace) with the Dowex^TM^ resin.

The mass spectrum of Fondaparinux prepared in water (blue trace) shows the presence of single-charged species including the intact compound [FDPX − 10H + 9Na]^−^ with a very low relative intensity compared to lighter-weight compounds with losses of sulfate groups. The pattern is complicated by the presence of various adducts. The spectrum therefore turns out to be quite complex for a single GAG molecule. On the contrary, the pretreatment of Fondaparinux on a Dowex^TM^ resin loaded with sodium ions simplifies the spectrum due to the presence of the majority sodium adduct and the presence of the intact compound, which is more intense compared to the initial spectrum obtained in water. The results therefore show that the use of an ion-exchange resin makes it possible to avoid almost all Na/K exchanges. Measurements of the proportion of forms with at least one Na/K exchange for each species (intact FDPX and desulfated species) are shown in Figure 2 (right). For each ion considered, the rate of exchange does not exceed 10% after pretreatment with the resin, whereas 70 to 90% of each species has exchanged at least one sodium ion with one potassium ion when the sample is not treated.

Pretreatment with Dowex^TM^ ion-exchange resin, as previously described [15], is therefore very effective in saturating an oligosaccharide such as Fondaparinux with a counterion of interest such as sodium in order to limit exchanges after deposition on the MALDI plate.

- On-line strategy for FDPX pretreatment at miniaturized scale,

In order to propose a preparation protocol on a miniaturized scale, the analysis of Fondaparinux by MALDI obtained by treatment on a capillary monolithic column (functionalized with sodium mercaptoethane sulfonate) previously saturated with sodium was carried out by depositing the solution directly at the capillary outlet on the MALDI plate (about 1 µL).

**Figure 3:**
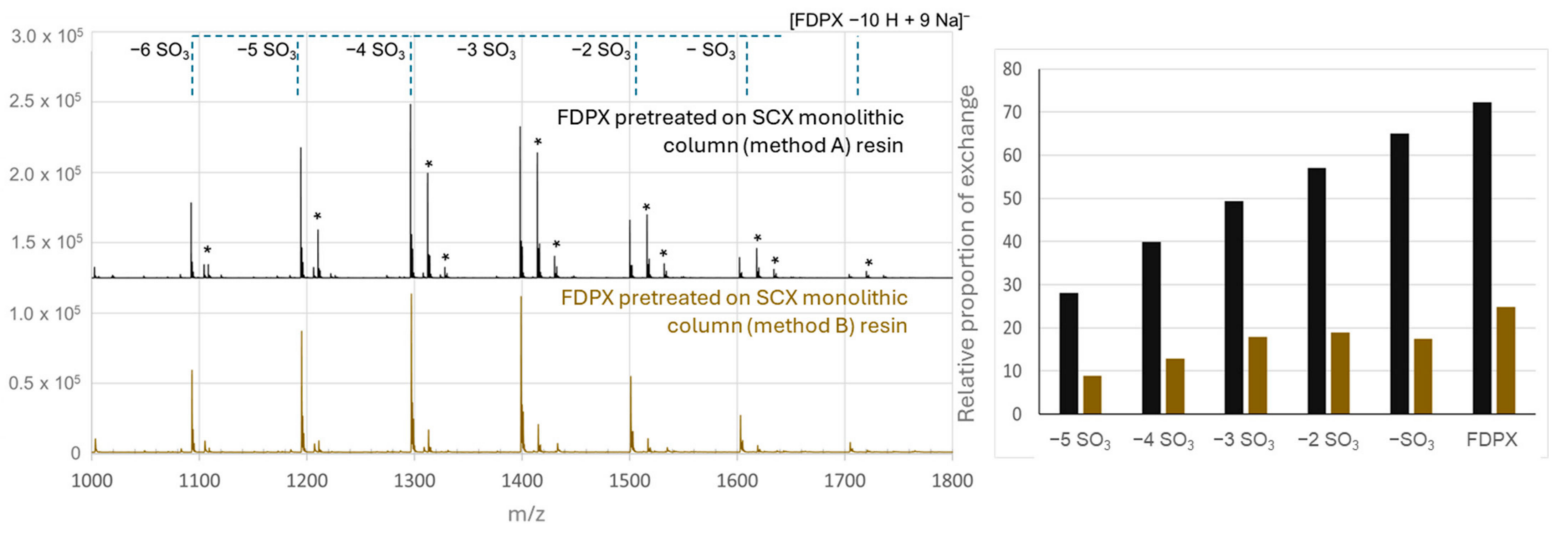
Mass spectra of Fondaparinux (50 µM) and proportion of forms with at least one Na/K exchange on the MALDI-TOF spectra (i) after pretreatment on a monolithic cation-exchange column (L = 8.5 cm, di = 75 µm) functionalized with sodium bisulfite and on-line elution on the MALDI plate (gray trace); (ii) after pretreatment on a monolithic cation-exchange column (L = 8.5 cm, di = 75 µm) functionalized with sodium mercaptoethane sulfonate and on-line elution on the MALDI plate (gold trace); MALDI-TOF-MS detection between 1000 and 1800 Th. The samples are prepared with a HABA/TMG ½ 80 mg/mL matrix in methanol at a ratio of 0.5/1 µL of matrix/sample on a MALDI Anchorship plate. The spectra were obtained with a laser power of 73% for 100,000 spectra. Asterisks represent Na/K exchanges.

The results are presented in Figure 3.

The results unexpectedly show that Fondaparinux pretreated on the monolithic cation-exchange columns is detected with an ionization state profile comparable to that of Fondaparinux prepared in untreated water, i.e., with a distribution of the signal over several sodium/potassium adducts, irrespective of the stationary phase. Similarly, desulfation phenomena are still present, with no particular effect of loading with sodium counter ion showing. The proportion of adducts present for each signal shows that 10 to 80% of Fondaparinux shows at least one Na/K exchange depending on the ion and the stationary phase considered. The monolithic ion-exchange column prepared from sodium bisulfite also shows the least efficient performance, with more Fondaparinux ions with at least one potassium counterion than initially present. The monolithic column prepared from SMES gives the best performance but does not completely prevent exchanges.

These results led us to evaluate the pretreatment of Fondaparinux with another commercial cation-exchange phase. Surprisingly, the use of this other type of cation-exchange column (SPE MCX column), despite its positive effect in nano-ESI-MS analysis, did not reduce either the Na/K exchanges or the number of signals for each compound in MALDI. Thus, none of the three stationary phases favored the full sodium forms of Fondaparinux to a greater extent in MALDI ionization, despite their beneficial effect when coupled on-line with nano-ESI. It therefore appears that Na/K exchanges occur between elution and MALDI-TOF analysis on the MALDI plate.

- Singularity of the results obtained with Dowex^TM^ resin

Based on the observation that the FDPX solutions were slightly colored after their contact with the Dowex^TM^ resin, and that peaks associated with a polymeric species constant mass difference of 106 *Th* were observed in the nano-ESI mass spectra (see Supplementary Material S2), the following hypothesis was put forward. After centrifugation of the Dowex^TM^ resin and collection of the supernatant, some residual resin (or contaminant) remains in the Fondaparinux solution. The presence of these elements could prevent the formation of various adducts on the MALDI plate during the analysis. This hypothesis would explain the difference observed between Dowex^TM^ resin treatment and ion exchange by chromatographic means (SCX monoliths).

**Figure 4:**
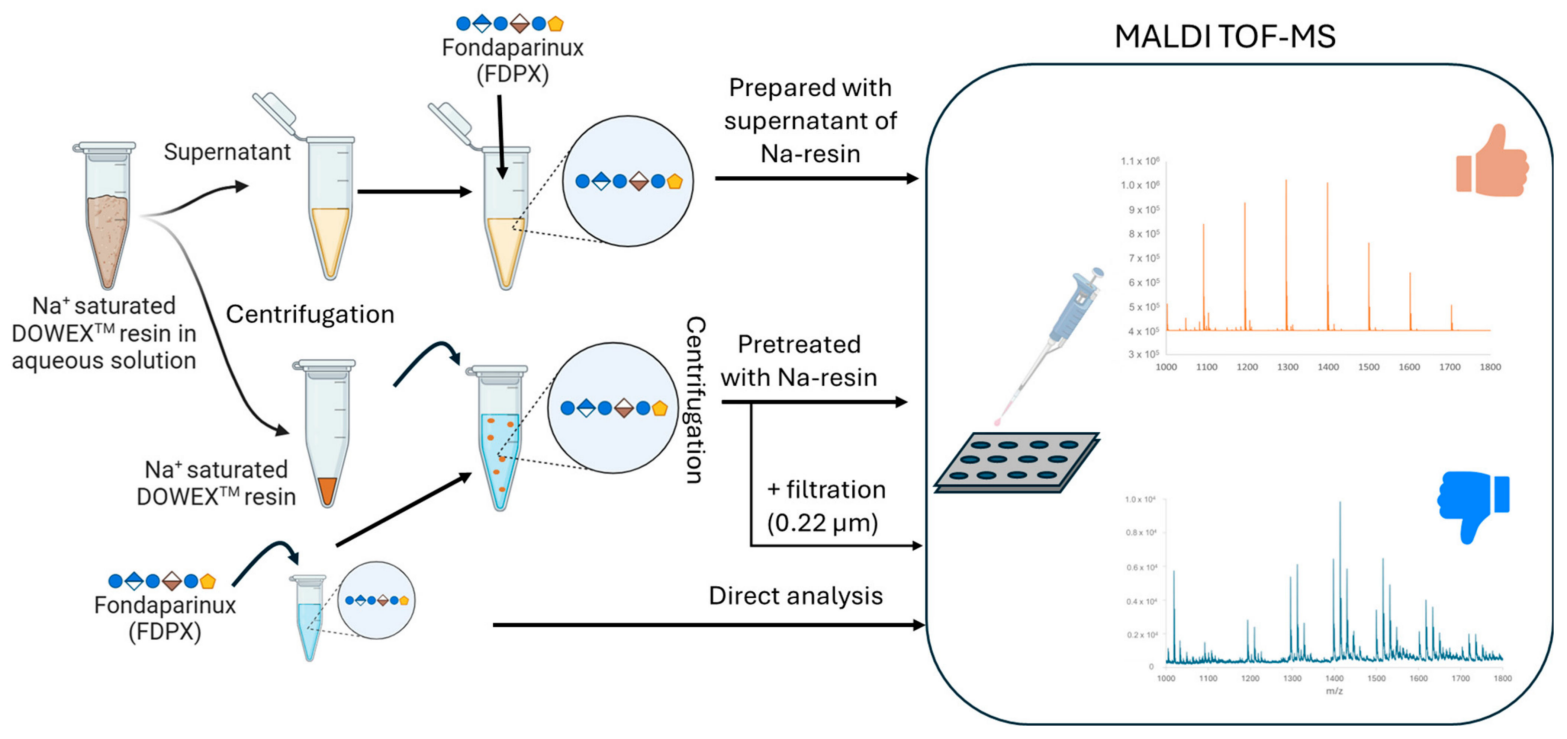
Schematic illustration of the experimental workflow of FDPX analysis by MALDI-TOF MS using DOWEX^TM^ resin.

A full characterization of the species present in the solution supernatant in contact with the Dowex^TM^ resin is presented in Supplementary Material S4 and has led to the following conclusion. After centrifugation, nanoparticles with functional groups close to those of the Dowex^TM^ resin (an aromatic ring, a sulphonate function, and a carboxylic acid function) are present in the supernatant. The increase of 106.0 *Th* observed on the mass spectrum suggests a polymeric form (ethylbenzene 106 *Th*). The impact of the presence of such resin residues in the solution on the quality of the MALDI mass spectra were subsequently investigated. To this end, a solution of Fondaparinux was prepared using the water supernatant from the Dowex^TM^ sodium-saturated resin (Figure 4).

**Figure 5:**
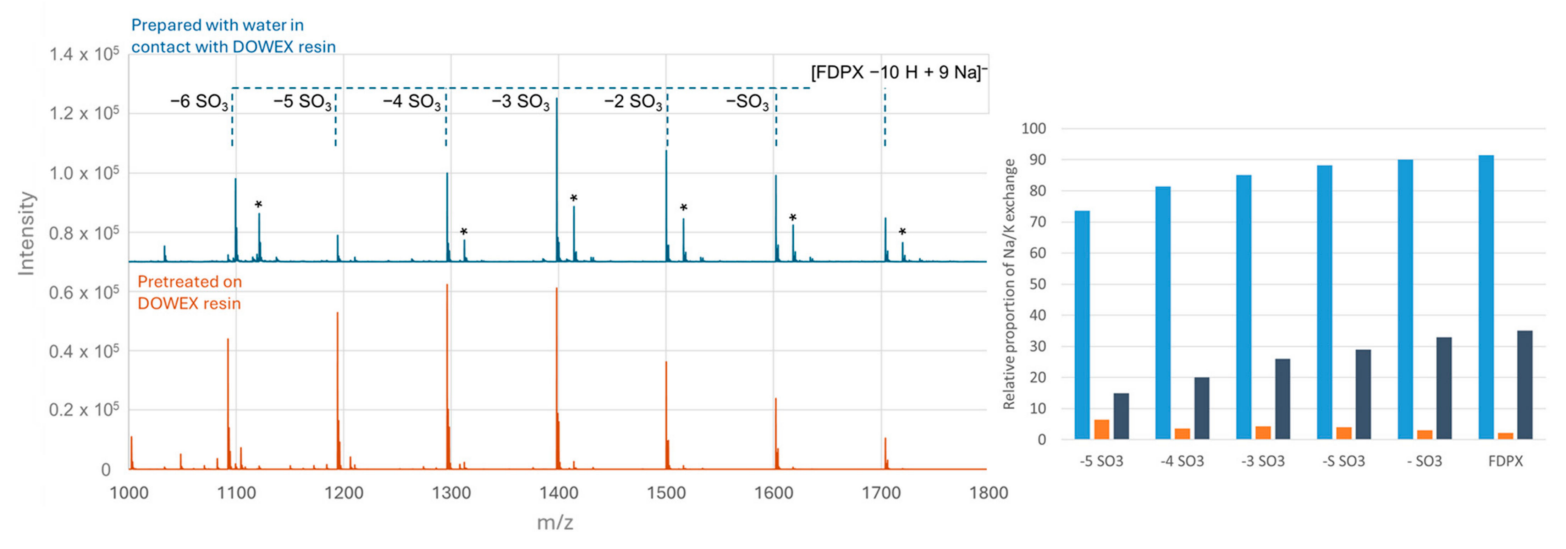
MALDI-TOF mass spectra of Fondaparinux (50 µM) centered between 1000 and 1800 Th (**left**) and proportion of forms presenting at least one Na/K exchange (**right**); (i) in water (blue); (ii) after treatment with the cation-exchange resin loaded with sodium (orange); (iii) in the supernatant of a mixture of water + resin converted to sodium (gray). The samples are prepared with a HABA/TMG ½ 80 mg/mL matrix in methanol at a ratio of 0.5/1 µL of matrix/sample on a MALDI Anchorship plate. The spectra were obtained with a laser power of 73% for 100,000 spectra. Asterisks represent Na/K exchanges.

The MALDI mass spectra obtained are shown in Figure 5. First of all, they show that after dilution in this supernatant, the FDPX is sodium saturated, as observed after a pretreatment with the resin.

Preparation of Fondaparinux in the supernatant resulted in a significant improvement, with only 30% or less of forms with at least one exchange observed and is a simple alternative to limit Na/K exchanges. However, although significant, this effect does not reach that obtained by pretreating the sample with the Dowex^TM^ converted resin directly.

Lastly, and to confirm our interpretation, it is worth noticing that filtering the Fondaparinux solution treated with the DOWEX^TM^ resin using a 0.22 µm filter to remove the particles from the solution before deposition on the MALDI suppresses the adducts’ suppression effects that are observed with this resin.

## 3. Materials and Methods

### 3.1. Reagents and Buffers

Sodium mercaptoethane sulfonate (SMES), Fondaparinux, (3-methacryloxypropyl)-trimethoxysilane (γ-MAPS), ethylene dimethacrylate (EDMA), glycidyl methacrylate (GMA), triethylamine (TEA), 1-propanol, 1,4-butanediol, dimethyl sulfoxide (DMSO), azobis(isobutyronitrile) (AIBN), 4-(4-Hydroxyphenylazo)benzoic acid (HABA), 1,1,3,3-tetramethylguanidine (TMG), and *DOWEX 50WX8 ion-exchange resin* were purchased from Sigma-Aldrich (L’Isle d’Abeau Chesne, France). All aqueous solutions were prepared using >18 MΩ deionized water. A phosphate buffer was prepared by dissolving 1.17 g of K_2_HPO4 in 100 mL of ultrapure water, and the pH was adjusted to 7.4 with phosphoric acid.

### 3.2. Monolithic Capillary Column Synthesis

Additionally, 75 µm i.d. fused-silica capillaries with polyimide (TSP) or with UV transparent coating (TSH) were purchased from Cluzeau info Labo (Sainte-Foy-La-Grande, France). Silica activation was performed by flushing 15 cm-length long capillaries with a 5% (*v*/*v*) solution of γ-MAPS in methanol/water (95/5, *v*/*v*) and 2.5% TEA for 1 h at 7 bars. The capillaries were then rinsed with methanol for 15 min at 7 bar and dried at room temperature under a nitrogen stream prior to use.

#### 3.2.1. In-Capillary Poly(GMA-co-EDMA) Monolith Synthesis

Poly(GMA-co-EDMA) monoliths were synthesized as described in a previous work. The polymerization mixture was prepared by mixing 0.9 mL GMA, 0.3 mL EDMA, 1.05 mL 1-propanol, 0.6 mL 1,4-butanediol, 0.15 mL ultrapure water, and 12 mg of AIBN initiator. The pretreated TSH capillary was filled with the polymerization mixture under 1 bar N_2_ pressure. The photopolymerization was performed in a Bio-link UV crosslinker (VWR International, France) under 365 nm UV light with a total energy of 6 J cm^−2^. A PEEK tube (380 μm i.d.) was used as a mask to cover non-irradiated areas. After polymerization, the capillary was rinsed with methanol for 1 h.

#### 3.2.2. Synthesis of Strong Cation-Exchange Monolithic Capillary Column Synthesis

Two functionalization pathways based on a poly(GMA-co-EDMA) monolithic column identified in the literature were tested; one from sodium bisulfite and the other from sodium mercaptoethane sulfonate (SMES) (Figure S2.2). These two methods have the advantage of being simple to implement and presenting the best performances in terms of cation-exchange capacity.

- Method A: Bisulfite method

The functionalization of the stationary phase is carried out according to the protocol described by Ueki et al. in 2004 [17]. A 1 M sodium bisulfite solution at pH 10 is percolated for 18 h at 75 °C under a pressure of 6 bars. The stationary phase is then rinsed for 2 h with a 15 mM HNO_3_ solution and then for 2 h with ultrapure water under a pressure of 6 bars.

- Method B: SMES method

The functionalization of the stationary phase is adapted from the protocol described by Percin et al. in 2015 [16]. A 0.1 M sodium 2-mercaptoethane sulfonate solution in 1 M NaOH medium is percolated for 18 h at 50 °C under a pressure of 6 bars. The stationary phase is then rinsed for 2 h with ultrapure water under a pressure of 6 bars.

#### 3.2.3. Evaluation of Cation-Exchange Capacity

The two prepared monolithic stationary phases were evaluated and compared through their cation-exchange capacities. For this, the total number of anionic sites was determined by the quantity of cations necessary to saturate the stationary phase. Firstly, the sulfonate groups are protonated by thoroughly flushing a 1 N HCl aqueous phase through the column. After a rinsing step with water, a 1 M NaCl solution is infused on the stationary phase. Online detection is carried out by conductimetry. Since the conductance of the Na^+^ ions is different from that of the K^+^ ions, a modification of the baseline signal is detected when the K^+^ ions breakthrough from the column. The time lapse enables determination of the cation-exchange capacity.

#### 3.2.4. Instrumentation

Capillary monolithic columns were prepared using a pressure gradient microfluidic flow controller MFCS-EZ100 system (Fluigent, Villejuif, France) to handle liquids inside the capillary. Inlet pressure values are reported to specify the liquid flow conditions (outlet pressure was always set up at atmospheric pressure). An LC pump L-6000 (Merck, Darmstadt, Germany) was used to rinse the freshly prepared monolithic capillary columns. Analysis of particle size distributions was conducted on zetasizer advance PRO red (Malvern, France).

#### 3.2.5. Nano-ESI Analysis

NanoLC-TOF-MS analyses were performed using a Nano-LC 400 system (Sciex, Villebon sur Yvette, France)connected to a TOF-MS analyzer (Agilent 6230 TOF LC/MS Waldbronn, Deutschland) with a nano-electrospray ionization source. An additional make-up flow (400 nL min^−1^) was added with the loading pump of the Nano-LC 400 system instrument and mixed with the chromatographic flow through a zero dead volume union (UH-750 from IDEX (Cluzeau info labo, France)). Nanospray stainless steel emitters with a uniform 30 μm inner diameter were purchased from Pepsep (Marslev, Denmark). This instrument set-up allows long term ion source stability using pure aqueous mobile phases. Experiments are conducted at room temperature using a 10 µL sample loop.

The use of strong cation-exchange monolithic capillary columns required, during the loading with cations, disconnection of the column from the MS detector to avoid the addition of NaCl in the ion source. The stationary phase is saturated with sodium cations by continuously equilibrating the column with a 1 M NaCl solution. After rinsing the column with water (5 column volumes), the MS detector is connected to the nano-LC instrument until the spray has stabilized. Finally, a 50 µM fondaparinux solution is percolated through the column and MS detector.

#### 3.2.6. MALDI Analysis

FDPX analysis after pretreatment on strong cation-exchange monolithic column. The stationary phase is first saturated with sodium cations by continuously balancing the column with a 1 M NaCl solution. After rinsing with water, a 50 µM Fondaparinux solution is percolated and then collected at the column outlet (or cartridge) before depositing 1 µL of solution on the matrix previously dried in open air. The co-crystallization is finally carried out in open air and under atmospheric pressure.

MALDI-TOFMS experiments were carried out with a MALDI-TOF MS (UltrafleXtreme from Bruker (Wissembourg, France) equipped with a 355 nm Smartbeam II laser with a 19 kV acceleration voltage and a 20 kV reflection voltage. MS data were acquired in the negative reflector ion mode and externally calibrated with a standard solution of Fondaparinux. Samples were deposited on a MTP AnchorChip target plate (from Bruker).

Experimental and calculated *m*/*z* values of detected MS ions in nanoLC-TOF MS (Table S4.2) and MALDI TOF-MS (Table S4.3) are presented in the Supporting Materials.

## 4. Conclusions

The in-line strategy (with ion-exchange monolithic columns) to control the ionization state of Fondaparinux in nano-ESI-MS detection is a straightforward method to limit signal dilution to multi-charged states and the loss of the fragile sulphate group. The full molecular species is visible for easy and direct molecular characterization. This strategy proved to be less interesting for MALD-TOF MS detection than the reference method using the Dowex^TM^ resin for Fondarapiux pretreatment. It turns out that some residues (nanoparticles of about 100 nm bearing aromatic rings, sulfate, and acidic functions) coming from the Dowex^TM^ resin are transferred to the MALDI plate with the Fondaparinux solution. These nanoparticles appear to protect Fondaparinux from Na/K exchange that can occur on the MALDI plate after sample deposition. Although less efficient than the pretreatment with the Dowex^TM^ resin, the use of water with nanoparticles acting as cation-exchanger can be used on the miniaturized scale to control the ionization state of GAG. Based on these observations, the production of well-characterized nanoparticles will be investigated to better control the GAG signature in MALDI-TOF MS and facilitate the interpretation of mass spectra of complex GAG mixtures such as low-molecular-weight heparin samples.

## Data Availability

Data are contained within this article and the Supplementary Materials.

## Supplementary Materials

The following supporting information can be downloaded at https://www.mdpi.com/article/10.3390/molecules29235642/s1: Supplementary Material S1: Mass spectrum of Fondaparinux in nanoESI-TOF and MALDI-TOF-MS. Supplementary Material S2: Mass spectrum of Fondaparinux in nano-ESI-MS with or without off-line sample pre-treatment with the sodium loaded or potassium loaded DowexTM resin. Supplementary Material S3: Strong cation exchange materials used for the pre-treatment of Fondaparinux. Supplementary Material S4: Characterization of suspended particles in the super-natant of DOWEXTM resin solutions.
