# Supplementary material for: Improved Detection of Polysulfated Oligosaccharides by Mass Spectrometry Applicable to Miniaturized Samples
Source: Molecules. 2024 Nov 28;29(23):5642. doi: 10.3390/molecules29235642 (PMC11643541; doi:10.3390/molecules29235642)
Supplement: Supplementary file 1 [file molecules-29-05642-s001.zip › molecules-3306402-supplementary-proofreading.pdf]

molecules

MDPI

Supplementary information

Improved detection of polysulfated oligosaccharides by mass spectrometry applicable to miniaturized samples

Frédéric Jeanroy [1], Julie Gil [1], Clothilde Comby-Zerbino [2], Claire Demesmay [1] and Vincent Dugas [1,*]

1 Université Claude Bernard Lyon 1, Institut des Sciences Analytiques, ISA UMR 5280, CNRS, 5 rue de la Doua, 69100 Villeurbanne, France; Frederic.jeanroy@univ-lyon1.fr; julie.gil@univ-lyon1.fr (J.G.); demesmay@univ-lyon1.fr (C.D.); vincent.dugas@univ-lyon1.fr (V.D.)
2 Université de Lyon, CNRS, Université Claude Bernard Lyon 1, CNRS, Institut Lumière Matière UMR 5306, F-69100, Villeurbanne, France; clothilde.zerbino@univ-lyon1.fr
* Correspondence: vincent.dugas@univ-lyon1.fr

Supplementary material S1 : Mass spectrum of Fondaparinux in nanoESI-TOF and MALDI-TOF-MS

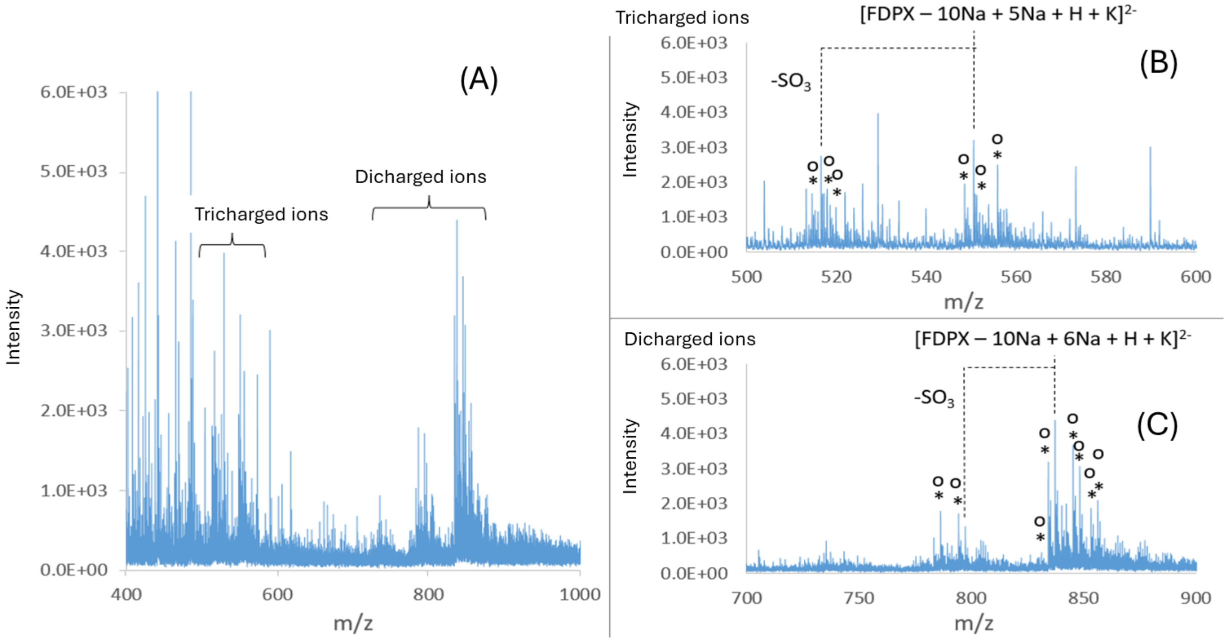

Figure S1.1: Mass spectrum of Fondaparinux (50 µM in water) obtained by nanoESI-TOF during infusion of the solution at 0.4 µL/min (**A**). Methanol at 0.4 µL/min is infused as an additional liquid. The spectra were obtained in negative polarity, at a source voltage of 1.4 kV and a fragmentor voltage of 300 V. Spectrum between *400* and *1000 Th* showing multiplycharged ions with a zoom on tricharged ions between *500* and *600 Th* (**B**) and a zoom on di-charged ions between *700* and *900 Th* (**C**). Asterisks represent Na/K exchanges and circles represent Na/H exchanges.

In the nanoESI-MS spectrum of Fondaparinux prepared at 50 µM in water and recorded in negative mode (Fig S1.1), it is possible to detect at least two different charged states (−3 and −2) and many adducts differing in the number of H, K or Na counterions.

In the spectrum of Fondaparinux prepared at 50 µM in water and recorded in negative mode in MALDI-TOF-MS (Fig S1.2), it is possible to detect at least two different charged states (−3 and −2) and many adducts differing in the number of H, K or Na counterions.

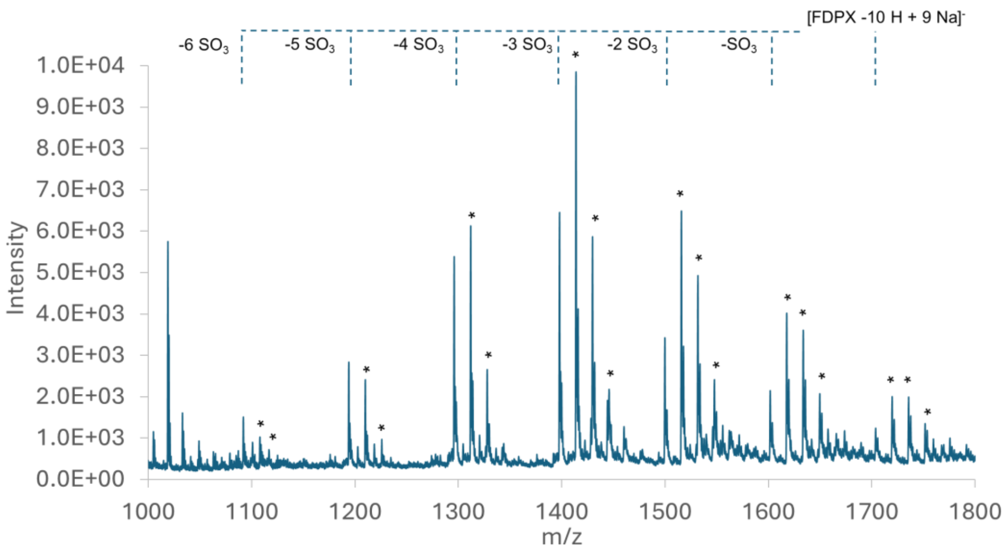

Figure S1.2: Mass spectra obtained by MALDI-TOF of Fondaparinux (50 µM). Samples are prepared with a HABA/TMG ½ 80 mg/mL matrix in methanol at a ratio of 0.5/1 µL of matrix/sample on a MALDI Anchorship plate. The spectra were obtained with a laser power of 60% for 100,000 spectra. Asterisks represent Na/K exchanges.

Supplementary material S2 : Mass spectrum of Fondaparinux in nano-ESI-MS with or without off-line sample pre-treatment with the sodium loaded or potassium loaded Dowex™ resin

After saturation with the cation of interest (Na^+ or K^+), the resin is added directly to the Fondaparinux solution. After centrifugation, the supernatant is collected and infused into the nano-ESI source. Figure S2.1 shows the mass spectra obtained with the sodium or potassium loaded resin.

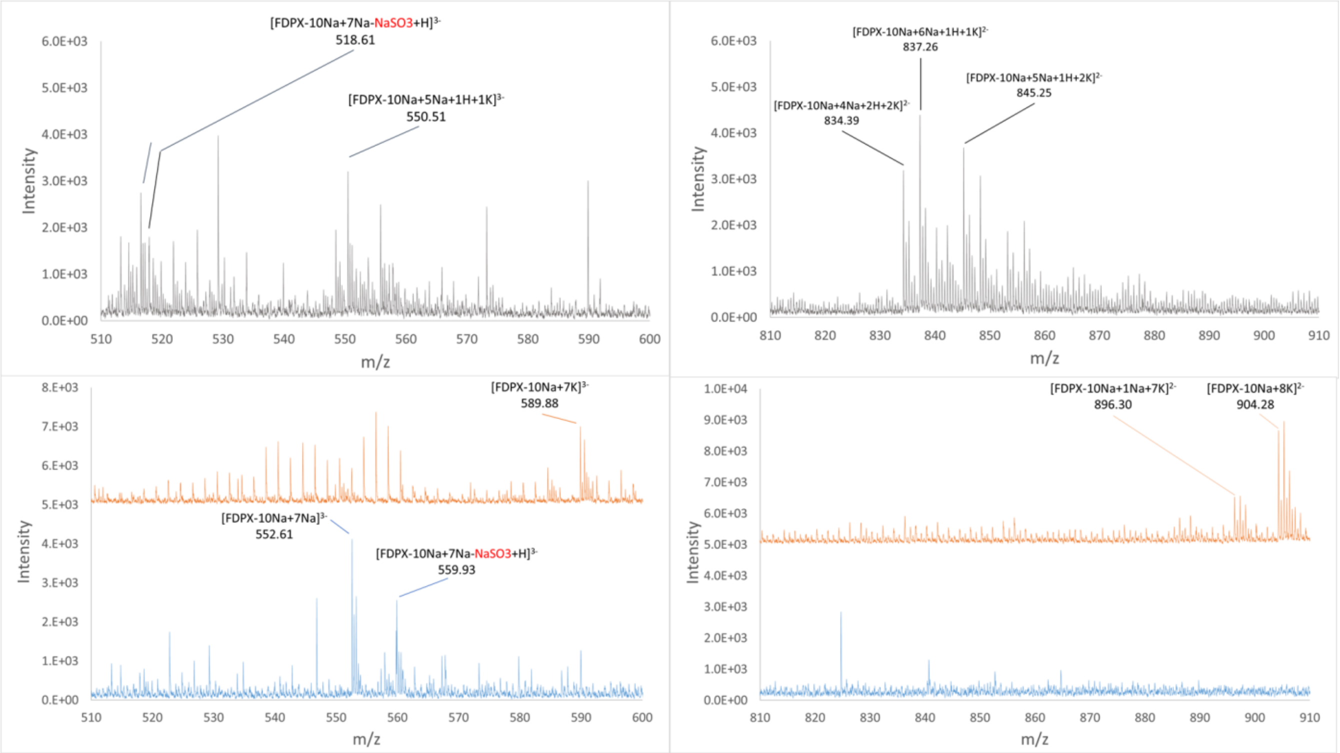

Figure S2.1: Mass spectra (nano-ESI (−)) of Fondaparinux prepared in water without pretreatment (grey plot) and after pretreatment with sodium loaded (blue plot) or potassium loaded Dowex™ resin (orange plot).

The off-line use of this Dowex cation exchange resin allows the vast majority of Fondaparinux to be converted with the counterion charged to the resin, as most forms are saturated with sodium or potassium. In addition, very few Na/K or Na/H exchanges are observed: no exchange is detected when the resin is charged with sodium and only one exchange (in the multiply charged forms) is observed when the resin is charged with potassium ions.

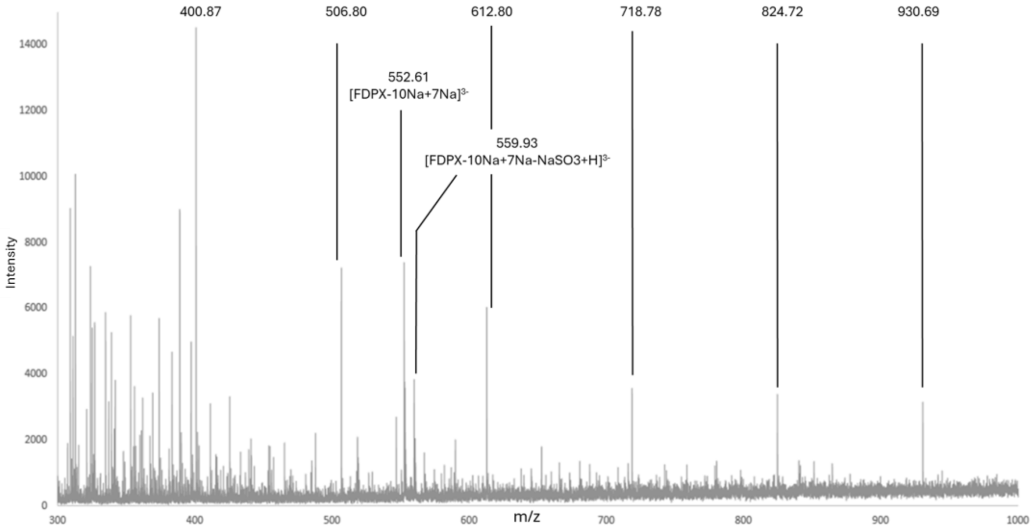

Figure S2.2: Mass spectra (ESI (−)) of Fondaparinux 50 µM prepared using water coming from the dowex™ resin supernatant.

Supplementary material S3 : Strong cation exchange materials used for the pretreatment of Fondaparinux

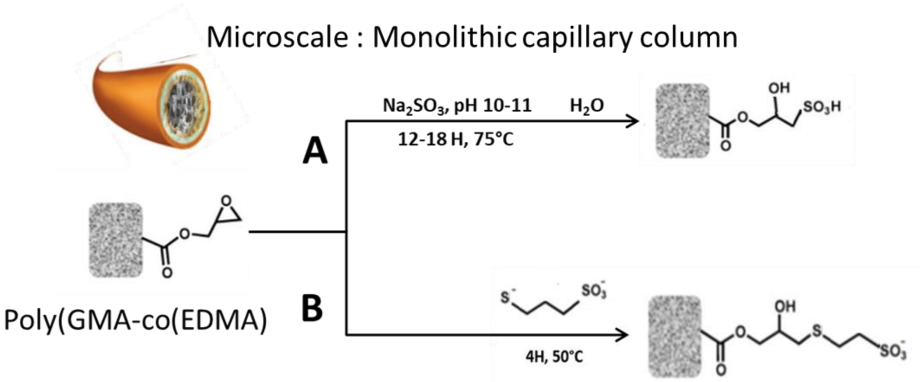

Figure S3: Strong cation exchange materials used for the pretreatment of Fondaparinux samples. Macroscale Dowex™ resin and microscale capillary column prepared from poly(GMA-co-EDMA) monolith using two preparation methods (Method A: modification of the epoxy ring by sodium bisulfite and method B: grafting of mercaptoethane sulfonate).

Supplementary material S4: Characterization of suspended particles in the supernatant of DOWEX™ resin solutions

Previous observations have suggested the presence of a compound in the supernatant that protects Fondaparinux from Na/K exchange. A UV-visible spectrometric analysis was carried out to confirm its presence/nature. A strong absorption is observed between 200 and 300 nm (Figure S4.1). This result confirms the presence of an unknown UV-absorbing compound in the supernatant. UV spectra (Lambda Bio-spectrophotometer (Perkin Elmer) was done using quartz cuvette with 1.0 cm path length.

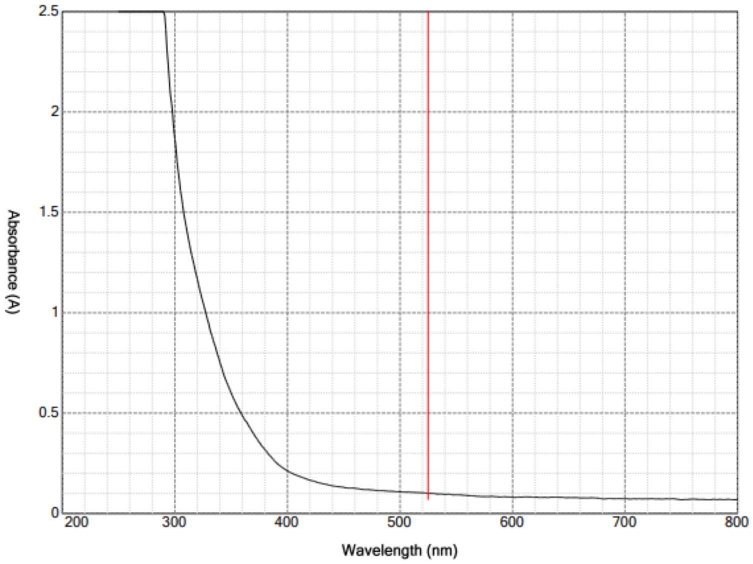

Figure S4.1: UV-visible spectrum of the supernatant from water placed in contact with a resin converted with Na^+ ions.

Characterization of the unknown compound by infrared spectroscopy and Raman spectroscopy

- Infrared spectra

Fourier transform infrared (FTIR) spectroscopy by NEXUS spectrophotometer was carried out to characterize the chemical characteristic of DOWEX™ resin and residue for supernatant. The spectra were recorded in attenuated total reflectance (ATR) mode with a Thunder dome (Spectra-tech) accessory containing germanium crystal with a mono reflection at 45°.

The infrared spectrum of the resin in the solid state is compared with the infrared spectra of the solid residue obtained from the evaporation of two supernatants: water placed in contact with the unconverted resin (with the H^+ counterion) and water placed in contact with the Na^+ converted resin.

The FTIR spectra shown in Figure S4.2 are very close to each other. Among the common absorption bands, it is possible to observe a broad band at 3437 cm^{-1} which may correspond to a stretching vibration of an O-H bond of an acid or alcohol function. Absorption bands corresponding to stretching vibrations of an S = 0 bond of a sulfonate SO_3 group are also visible at 1201, 1026 and 1005 cm^{-1}. Bands at 831 cm^{-1} and 760 cm^{-1} observed for the unconverted resin in powder are also detected. These bands may correspond to deformation vibrations of C-H bonds associated with a disubstituted aromatic cycle. These two bands are also detected in the two supernatants but the signal at 760 cm^{-1} is very weak. The water in contact with the resin converted to sodium shows both bands. The interpretation of the absorption bands is summarized in Table S4.3.

The supernatant of the water in contact with the resin converted to Na shows a significant signal between 1500 cm^{-1} and 1300 cm^{-1} which is not detected on the other spectra. This signal is not due to the resin but to the methanol used to dry the resin after conversion to sodium. It is therefore ignored, when interpreting the spectra.

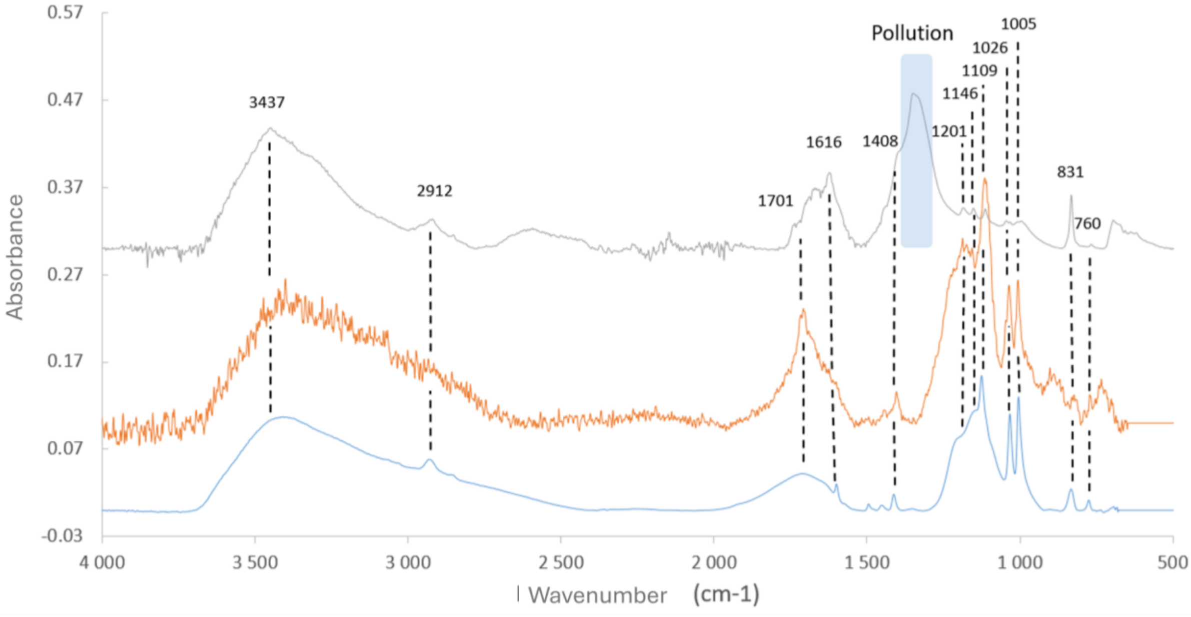

Figure S4.2: Fourier transform infrared spectra of the cation exchange resin in powder form before conversion with sodium (blue line), of the supernatant obtained after centrifugation at 3000 rpm for 3 min of water + unconverted resin (orange line) and of the supernatant obtained after centrifugation at 3000 rpm for 3 min of water + resin converted using sodium (gray line). The IR spectra of the two supernatants are obtained after drying.

Table S4.1: Interpretation of the absorption bands observed on the FTIR spectra (i) of the cation exchange resin in powder form before conversion to sodium, (ii) of the supernatant obtained after centrifugation at 3000 rpm for 3 min of water + unconverted resin and (iii) of the supernatant obtained after centrifugation at 3000 rpm for 3 min of water + resin converted to sodium.

Wavenumber (cm^{-1})	Chemical bond	Vibration modes	Chemical function
760	δC-H	bending	Disubstituted aromatic
831	δC-H	bending	Disubstituted aromatic
1005	δC-H	In-plane bending	Aromatic core
1026	νS=O	Symmetric stretching	SO_3
1109	νS=O	Symmetric stretching	SO_3
1146	νS=O	Symmetric stretching	SO_3
1184	νS=O	Asymmetric stretching	SO_3
1616	δC=C	bending	Aromatic core
2912	νO-H	stretching	SO_3
3437	νO-H	stretching	Alcool / acid

- Raman spectra

The Raman spectrum of the supernatant in contact with the resin converted into Na (Figure S4.3) also confirms the presence of a compound with an O-H bond with two characteristic bands at 3168 cm^{-1} and 1648 cm^{-1}. These two bands may in fact correspond to an stretching vibration of an O-H bond and to an asymmetric stretching vibration of a C=O bond respectively.

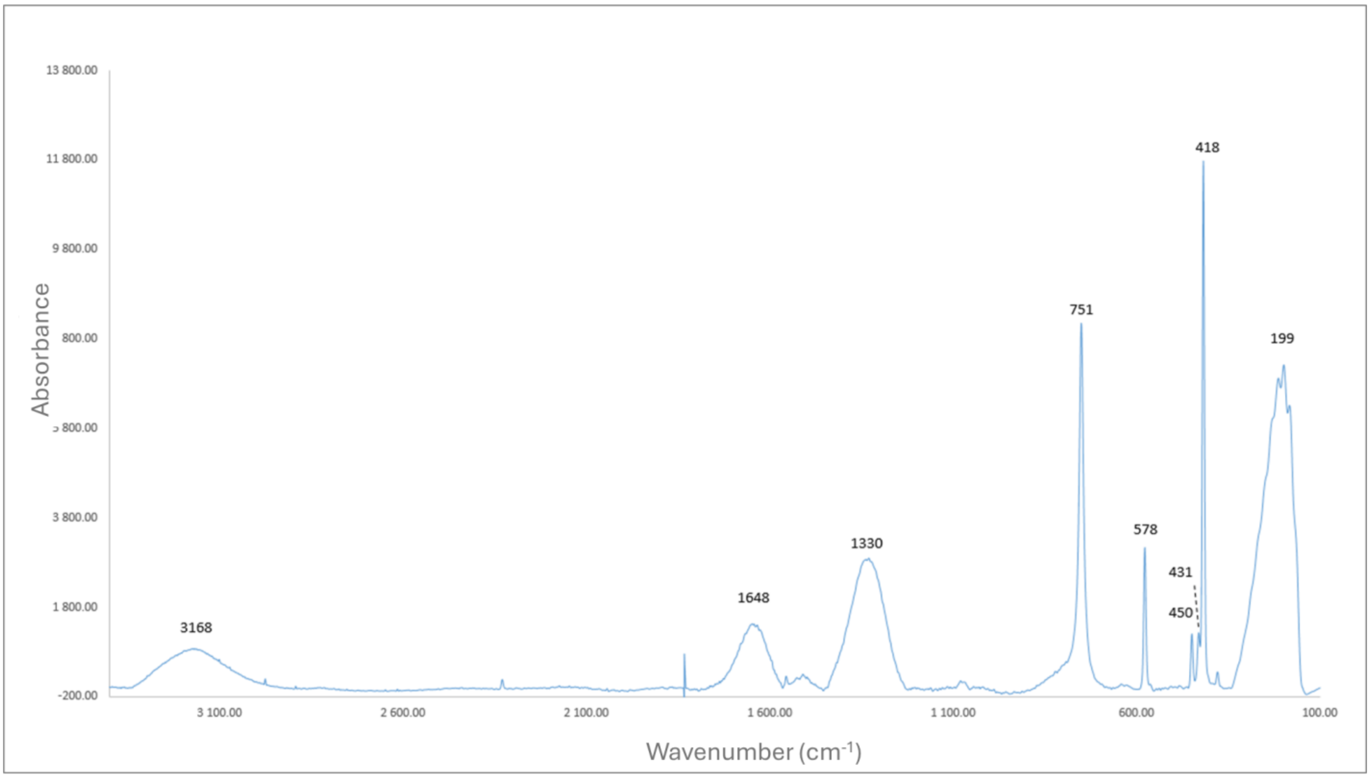

Figure S4.3: Raman spectrum of the supernatant obtained after centrifugation at 3000 rpm for 3 min of water + resin converted to sodium.

- Mass spectra

The mass spectra of the supernatants of the mixture of water and resin converted or not (Figure S4.4) show signals with a constant mass difference of *106.0 Th* with a nominal m/z ratio of *400.8 Th*. These signals also correspond to the signals observed during the nanoESI-TOF analysis of FDPX after treatment with the resin converted to sodium and not previously identified. The charge state could not be determined due to the absence of isotopes detected at M+1. However, these compounds are not detected in the supernatant when the resin has not been converted to Na.

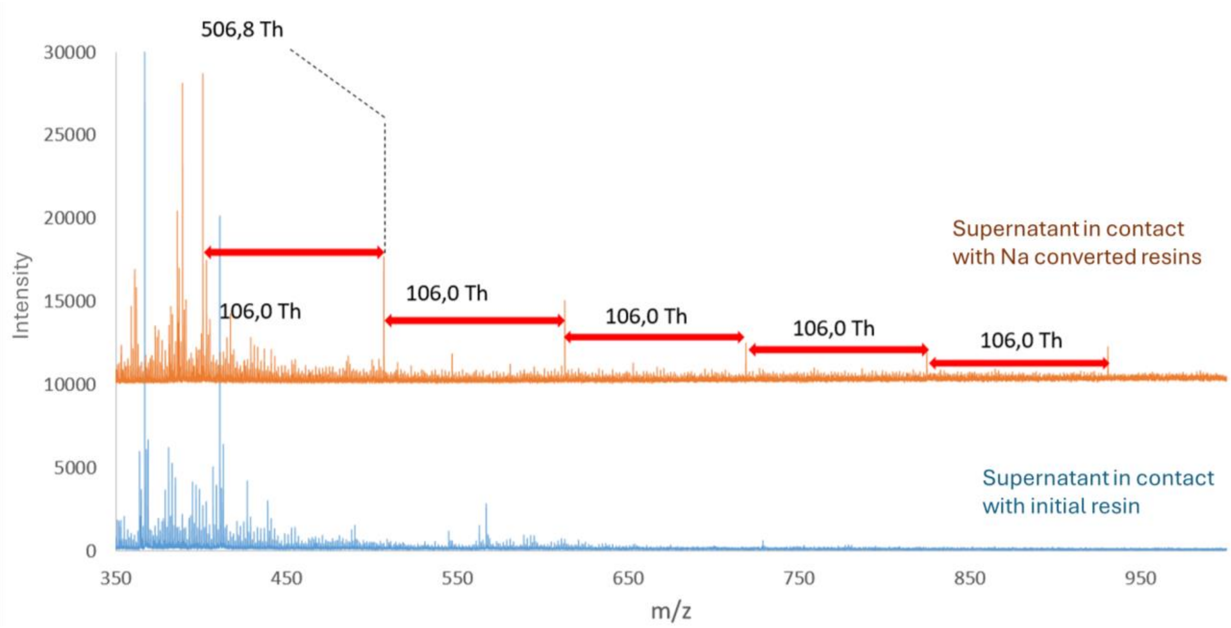

Figure S4.4: Mass spectra of supernatants are from a mixture of water and unconverted resin (blue line) and a mixture of water and sodium converted resin (orange line). The spectra were obtained in negative polarity, at a source voltage of 1.4 kV and a fragmentor voltage of 300 V. Spectra between *400* and *1000 Th* showing multiply charged ions. The asterisks represent Na/K exchanges and the circles represent Na/H exchanges.

Finally, it should be noted that when using the resin converted to Na^+, there are single charged ions separated by an interval of *106 Th* (Figure S2.2). These ions were not identified as fragments of Fondaparinux. However, these regular intervals may indicate the presence of a polymeric compound in the supernatant

- Light scattering analysis

A light scattering analysis (Zetasizer advance PRO red (Malvern Panalytical)) also revealed the presence of nanoparticles of around 100 nm in the supernatant, much smaller than the particles contained in the resin (100–200 µm).

All of these results therefore suggest the presence of nanoparticles with functional groups close to those of the DOWEX resin : an aromatic ring, a sulfonate function and a carboxylic acid function. The increment of *106.0 Th* observed on the mass spectrum suggests a polymeric form (ethylbenzene *106 Th*).

Table S1. Main characteristic ions of Fondaparinux obtained in negative polarity mode with a source voltage of –1.4 kV and a fragmentor voltage of 300 V.

m/z	Charge	Identification	Theoretical m/z
512.52	–2	[FDPX – 10Na + 8H – 6SO3]2–	512.60
552.48	–2	[FDPX– 10Na + 8H – 5SO3]2–	552.58
592.46	–2	[FDPX– 10Na + 8H – 4SO3]2–	592.55
632.43	–2	[FDPX – 10Na + 8H – 3SO3]2–	632.53
672.41	–2	[FDPX – 10Na + 8H – 2SO3]2–	672.51
712.38	–2	[FDPX – 10Na + 8H – SO3]2–	712.49
752.35	–2	[FDPX – 10Na + 8H]2–	752.47
783.29	–2	[FDPX – 10Na + 3Na + 3H + 2K – SO3]2–	783.42
786.30	–2	[FDPX – 10Na + 5Na + 2H + K – SO3]2–	786.42
791.31	–2	[FDPX – 10Na + 2Na + 3H + 2K – SO3]2–	791.40
794.29	–2	[FDPX – 10Na + 4Na + 2H + 2K – SO3]2–	794.41
797.30	–2	[FDPX – 10Na + 6Na + H + K – SO3]2–	797.41
831.25	–2	[FDPX – 10Na + 2Na + 3H + 3K]2–	831.38
834.25	–2	[FDPX – 10Na+4 Na + 2K + 2H]2–	834.39
837.26	–2	[FDPX – 10Na+6 Na + 1K + H]2–	837.39
840.27	–2	[FDPX – 10Na+8 Na]2	840.40
842.25	–2	[FDPX – 10Na + 3Na + 2H + 3K]2–	842.37
845.25	–2	[FDPX – 10Na + 5Na + H + 2K]2–	845.38
848.27	–2	[FDPX – 10Na+7 Na + 1K]2	848.38
853.27	–2	[FDPX – 10Na + 4Na + H + 3K]2–	853.37
869.38	–2	[FDPX – 10Na+2 Na + 5K]2	869.34
514.53	–3	[FDPX – 10Na + 2Na + 3H + 2K – SO3]3–	514.61
516.53	–3	[FDPX – 10Na + 2H + 4Na + 1K – SO3]3–	516.62
519.86	–3	[FDPX – 10Na + Na + 3H + 3K – SO3]3–	519.94
521.85	–3	[FDPX – 10Na + 3Na + 2H + 2K – SO3]3–	521.94
529.19	–3	[FDPX – 10Na + 4Na + H + 2K – SO3]3–	529.27
548.51	–3	[FDPX – 10Na + 3Na + 2H + 2K]3–	548.60
552.61	–3	[FDPX – 10Na + 7Na]3	552.60
553.81	–3	[FDPX – 10Na + 2Na + 3K + 2H]3–	553.92
555.81	–3	[FDPX – 10Na +4 Na + 2K + H]3–	555.92

557.87	–3	[FDPX – 10Na + 6Na + K]3–	557.92
565.82	–3	[FDPX – 10Na +2 Na + 4K + H]3–	556.57
573.23	–3	[FDPX – 10Na +3 Na + 4K]3	573.90

Table S2. Ion identification of MADI-MS spectrum of Fondaparinux at 50 pmol per spot. Samples were prepared with HABA/TMG ½ 80 mg mL^{-1} in methanol matrix and a ratio 0.5/1 μL of matrix/sample was deposited on AnchorChip plate.

m/z	Charge	Identification	Theoretical m/z
1092.19	–1	[FDPX – 10Na + 3 Na + 6H – 6SO3]–	1092.15
1108.183	–1	[FDPX – 10Na + 2 Na + K + 6H – 6SO3]–	1108.12
1124.111	–1	[FDPX – 10Na + 1 Na + 2K + 6H – 6SO3]–	1124.1
1194.14	–1	[FDPX – 10Na + 4 Na + 5H – 5SO3]–	1194.09
1210.13	–1	[FDPX – 10Na + 3 Na + K + 5H – 5SO3]–	1210.06
1226.06	–1	[FDPX – 10Na + 2 Na + 2 K + 5H – 5SO3]–	1226.04
1242.049	–1	[FDPX – 10Na + 1 Na + 3 K + 5H – 5SO3]–	1226.04
1296.09	–1	[FDPX – 10Na + 5 Na + 4H – 4SO3]–	1296.03
1312.01	–1	[FDPX – 10Na + 4 Na + K + 4H – 4SO3]–	1312.00
1328.06	–1	[FDPX – 10Na + 3 Na + 2 K + 4H – 4SO3]–	13280.6
1343.99	–1	[FDPX – 10Na + 2 Na + 3 K + 4H – 4SO3]–	1343.95
1398.04	–1	[FDPX – 10Na + 6 Na + 3H – 3SO3]–	1397.96
1413.98	–1	[FDPX – 10Na + 5 Na + 1 K + 3H – 3SO3]–	1413.94
1429.99	–1	[FDPX – 10Na + 4 Na + 2 K + 3H – 3SO3]–	1429.91
1445.92	–1	[FDPX – 10Na + 3 Na + 3 K + 3H – 3SO3]–	1445.89
1461.90	–1	[FDPX – 10Na + 2 Na + 4 K + 3H – 3SO3]–	1461.86
1500.03	–1	[FDPX – 10Na +7 Na+ 2H – 2SO3]–	1499.90
1515.89	–1	[FDPX – 10Na + 6 Na + 1 K + 2H – SO3]–	1515.88
1531.95	–1	[FDPX – 10Na + 5 Na + 2 K + 2H – 2SO3]–	1531.85
1547.87	–1	[FDPX – 10Na + 4 Na + 3 K + 2H – 2SO3]–	1547.83
1569.87	–1	[FDPX – 10Na + 3 Na + 4 K + 2H – 2SO3]–	1569.81
1601.99	–1	[FDPX – 10Na +8 Na+ H – SO3]–	1601.84
1617.88	–1	[FDPX – 10Na +7 Na+ 1 K + H – SO3]–	1617.82
1633.92	–1	[FDPX – 10Na +6 Na+ 2 K + H – SO3]–	1633.79
1649.83	–1	[FDPX – 10Na +5 Na+ 3 K + H – SO3]–	1649.76
1670.64	–1	[FDPX – 10Na +4 Na+ 4 K + H – SO3]–	1669.78
1703.89	–1	[FDPX – 10Na +9 Na]–	1703.78
1717.99	–1	[FDPX – 10Na + Na + 5 K + 3H]–	1717.70
1733.77	–1	[FDPX – 10Na + 6 K + 3H]–	1733.68
1755.77	–1	[FDPX – 10Na + Na + 6 K + 2H]–	1755.66
